# “Large eaters” meet blood vessels: a new thematic series on macrophages and angiogenesis

**DOI:** 10.1186/2045-824X-4-17

**Published:** 2012-10-22

**Authors:** Jan Kitajewski

**Affiliations:** 1 Pathology, OB/GYN, and Herbert Irving Comprehensive Cancer Center, Columbia University Medical Center, 1130 St. Nicholas Ave, NY, NY, 10032, USA

## Abstract

*Vascular Cell* has launched a new series on macrophages and angiogenesis, a quickly evolving field critical to blood and lymphatic vessels during development, inflammation and tumorigenesis.

## Editorial

The term “macrophage” comes from the Greek for “large eaters”, a name that reflects the prominent phagocytic activity. Arising from the embryo, yolk sac, or the bone marrow, macrophages are most widely appreciated as critical members of the inflammatory response to pathogens; however, their close physical association with blood vessels has earned them increased attention from angiogenic researchers. Recent studies have established that macrophages influence blood vessel development during growth, tissue repair, and tumorigenesis, and their widespread contributions to angiogenesis are only beginning to be understood.

*Vascular Cell* presents a new thematic series entitled “Macrophages and Angiogenesis”, that provides reviews and analyses from experts in this emerging field. The functions of macrophages are as numerous as the names given to these myeloid-derived cells, and while the general focus here is on macrophages, the individual reviews range far and wide in their consideration of myeloid-derived cells involved in angiogenesis. The three initial articles of the series review the potential angiogenic activities of inflammatory macrophages, resident macrophages, microglia, retinal myeloid cells, dendritic cells, myeloid-derived suppressor cells, and more. A range of blood vessel types are considered, with discussion of developmental vessels, adult tissue vessels, tumor vessels and lymphatic vessels.

Natasha Harvey and Emma Gordon consider the interface between macrophages and lymphangiogenesis in their review entitled, “Deciphering the roles of macrophages in developmental and inflammation stimulated lymphangiogenesis” [1]. The authors consider the developmental origins of macrophages in relation to the development of the lymphatic vascular system, and discuss the intimate relationship between macrophages and lymphatic endothelial cells. They provide an excellent description of how macrophages guide and grow the lymphatic vasculature, and the mechanisms involved in this interaction. Both lymphatics and macrophages are key to the inflammatory response, and the authors describe the ways in which they work together to mediate both host responses during inflammation and lymphatic vessel growth. They extend their analysis to a discussion of tumor lymphangiogenesis, which highlights the key roles for macrophages during this pathological process. In closing, the authors synthesize an elegant model to highlight and consider the diverse roles of macrophages in developmental, inflammatory, and tumor lymphangiogenesis.

The article by Andrew Newman and Christopher Hughes, entitled, “Macrophages and angiogenesis: a role for Wnt signaling” [2], explores the roles for Wnt signaling molecules in macrophage biology, with a focus on angiogenic regulation. Wnts are key morphogens and mitogens, and among their diverse functions are the abilities to both promote blood vessel growth and to drive their regression. A thorough introduction to Wnt function in angiogenesis is followed by an outline of the evidence that macrophages are critical angiogenic agents. This focus on pro-angiogenic activities highlights Wnt-5a, which regulates angiogenesis by both direct and indirect mechanisms. They next discuss Wnt signaling effector proteins that mediate the angiogenic response to macrophages, including matrix metallo-proteinases (MMPs) and the endothelial receptor tyrosine kinase, Tie-2. The authors highlight recent elegant studies of macrophages in the eye, where they are critical for both regressing hyaloid vessels and for patterning retinal vasculature. The article closes with a model that considers how Wnts integrate these diverse angiogenic activities, with special attention paid to chemotactic cytokines released by macrophages.

The tumor microenvironment is full of myeloid-derived cells, and this is the topic covered by Michael Schmid and Judith Varner in their contribution entitled, “Myeloid cells in tumor inflammation” [3]. The concept of a tumor as a “wound that never heals”, posited by Harold Dvorak [4], highlights the intense inflammatory response that arises during tumorigenesis. Schmid and Varner describe the myeloid lineage cells involved in tumor inflammation and angiogenesis, and provide a guide to the surface markers that distinguish the key cell types. They pay special attention to myeloid-derived suppressor cells, which are the immunosuppressive cells that inhibit innate and adaptive immunity to promote tumor immune escape. Tumor cells secrete a diverse array of cytokines, and the authors discuss the cytokines that are involved in recruitment of tumor-associated macrophages. They additionally describe the mechanisms by which integrins function to recruit myeloid cells to the tumor microenvironment, and review the studies that have demonstrated this link. How macrophages may promote tumor angiogenesis and metastasis is a key element of this comprehensive analysis, and of particular interest is a discussion of how myeloid cells function in relapse or resistance to anti-tumor therapies. These authors synthesize several complex processes, involving diversity of myeloid cells and the tumor environment, into a series of well-crafted descriptions of the players, the processes, and implications for therapy.

More contributions are on their way, and we encourage experts in the field to contribute their analyses to this series. To submit your manuscript, please use the online submission system for *Vascular Cell* and indicate in your cover letter that you would like it to be considered for this thematic series. Or, please send a pre-submission enquiry to jkk9@columbia.edu and join this venue to teach and learn about macrophages in angiogenesis.
